# Acute Liver Failure Induces Glial Reactivity, Oxidative Stress and Impairs Brain Energy Metabolism in Rats

**DOI:** 10.3389/fnmol.2019.00327

**Published:** 2020-01-10

**Authors:** Pedro Arend Guazzelli, Giordano Fabricio Cittolin-Santos, Leo Anderson Meira-Martins, Mateus Grings, Yasmine Nonose, Gabriel S. Lazzarotto, Daniela Nogara, Jussemara S. da Silva, Fernanda U. Fontella, Moacir Wajner, Guilhian Leipnitz, Diogo O. Souza, Adriano Martimbianco de Assis

**Affiliations:** ^1^Post-graduate Program in Biological Sciences: Biochemistry, ICBS, Universidade Federal do Rio Grande do Sul—UFRGS, Porto Alegre, Brazil; ^2^Department of Biochemistry, Universidade Federal do Rio Grande do Sul—UFRGS, Porto Alegre, Brazil; ^3^Post-graduate Program in Health and Behavior, Health Sciences Centre, Universidade Católica de Pelotas—UCPel, Pelotas, Brazil

**Keywords:** acute liver failure, brain energy metabolism, hepatic encephalopathy, redox homeostasis, mitochondria, glial reactivity

## Abstract

Acute liver failure (ALF) implies a severe and rapid liver dysfunction that leads to impaired liver metabolism and hepatic encephalopathy (HE). Recent studies have suggested that several brain alterations such as astrocytic dysfunction and energy metabolism impairment may synergistically interact, playing a role in the development of HE. The purpose of the present study is to investigate early alterations in redox status, energy metabolism and astrocytic reactivity of rats submitted to ALF. Adult male Wistar rats were submitted either to subtotal hepatectomy (92% of liver mass) or sham operation to induce ALF. Twenty-four hours after the surgery, animals with ALF presented higher plasmatic levels of ammonia, lactate, ALT and AST and lower levels of glucose than the animals in the sham group. Animals with ALF presented several astrocytic morphological alterations indicating astrocytic reactivity. The ALF group also presented higher mitochondrial oxygen consumption, higher enzymatic activity and higher ATP levels in the brain (frontoparietal cortex). Moreover, ALF induced an increase in glutamate oxidation concomitant with a decrease in glucose and lactate oxidation. The increase in brain energy metabolism caused by astrocytic reactivity resulted in augmented levels of reactive oxygen species (ROS) and Poly [ADP-ribose] polymerase 1 (PARP1) and a decreased activity of the enzymes superoxide dismutase and glutathione peroxidase (GSH-Px). These findings suggest that in the early stages of ALF the brain presents a hypermetabolic state, oxidative stress and astrocytic reactivity, which could be in part sustained by an increase in mitochondrial oxidation of glutamate.

## Introduction

Acute liver failure (ALF) is a syndrome characterized by sudden hepatic injury and dysfunction in patients with a previously healthy liver and is associated with high lethality and morbidity (Craig et al., 2010; Bernal, 2017). The characteristic features of this condition are impaired liver synthetic function (expressed as coagulopathy), hepatic encephalopathy (HE) and, in severe cases, multi-organ failure (Craig et al., 2010; Scott et al., 2013). The manifestation of the neurological impairment under ALF varies from minor confusion, disorientation and sleep disorders to severe agitation, delirium and, in most advanced stages, coma and death (Blei and Larsen, 1999; Bernal, 2017). Indeed, the final stages of HE reach 20–25% of lethality due to cerebral edema and high intracranial pressure (Larsen and Wendon, 2008; Stravitz and Larsen, 2009) which demands rapid and aggressive treatment strategies such as liver transplantation (Acharya and Bajaj, 2018; Rajaram and Subramanian, 2018).

The molecular basis involved in the development of HE is complex and still a matter of debate and controversy. Nonetheless, ammonia appears to be the main factor in the progress of this syndrome (Bjerring et al., 2009; Hadjihambi et al., 2018). Ammonia is mostly metabolized in the liver *via* the urea cycle, and thus, ammonia bloodstream levels increase in the context of liver insufficiency (Bjerring et al., 2009; Scott et al., 2013). Ammonia can cross the blood-brain barrier by diffusion (Cooper et al., 1985) and numerous studies have shown a positive correlation between its arterial concentration and intracranial hypertension in humans (Clemmesen et al., 1999; Bernal et al., 2007).

Astrocytes occupy around one-third of the cerebral cortex volume and are involved in various neurochemical and cellular regulatory processes (Souza et al., 2019), including AFL (Scott et al., 2013). Astrocytes are the only brain cells that contain glutamine synthetase (GS), an essential enzyme of the glutamatergic system. Therefore, when ammonia concentration increases in the brain, these glial cells start to detoxify it by converting glutamate to glutamine catalyzed by GS (Martinez-Hernandez et al., 1977). Albrecht and Norenberg (2006) proposed the “Trojan Horse” hypothesis which suggests that glutamine works as a carrier of ammonia into the astrocytes’ mitochondria once it is metabolized back to glutamate and ammonium, leading to oxidative stress and cell dysfunction (Albrecht and Norenberg, 2006).

Previous studies demonstrated that cultured astrocytes treated with ammonia increased reactive oxygen species (ROS) levels, such as superoxide (Murthy et al., 2001), and the same effect was seen in a hyperammonemia rat model (Kosenko et al., 1997) and clinical studies (Montes-Cortes et al., 2019). Another study showed that ammonia increased mRNA levels of heme-oxygenase-1 (HO-1)—a typical marker of oxidative stress—in rats with HE (Warskulat et al., 2002). Furthermore, the administration of antioxidants such as vitamin E, catalase (CAT), and superoxide dismutase (Ulm et al., 2007) reduced ammonia-induced astrocyte swelling in rats (Jayakumar et al., 2006).

Oxidative stress is known to induce mitochondrial permeability transition (Crompton et al., 1987), which then causes the opening of the permeability transition pore (PTP), a non-selective channel in the inner mitochondrial membrane. The PTP leads to swelling of the mitochondrial matrix, defective adenosine triphosphate (ATP) production, and oxidative phosphorylation, increasing the formation of free radicals and creating a vicious cycle that results in cellular dysfunction (Zoratti et al., 2005). Furthermore, hyperammonemia has been reported to impair energy metabolism not only due to PTP but also directly affecting enzymes involved in energy metabolism (Heidari, 2019). In this regard, previous studies demonstrated that ammonia inhibits α-ketoglutarate dehydrogenase (α-KGDH) and isocitrate dehydrogenase activities (Walker, 2014) and decreases oxygen consumption in the brain (Alman et al., 1956; Strauss et al., 2003; Iversen et al., 2009; Dam et al., 2013). Nonetheless, studies with animal models of ALF have shown that brain ATP levels were only moderately decreased and the TCA cycle was not inhibited under acute HE (Hindfelt and Siesjö, 1971; Hindfelt et al., 1977), implying that the effects of hyperammonemia on brain energy metabolism are still a matter of debate. Considering the above stated, it is urgent to expand the knowledge regarding the mechanisms that lead to astrocyte dysfunction in acute HE in order to establish innovative therapeutic strategies.

The surgical resection of the liver is a well-established and extensively studied animal model of ALF and presents the fundamental features of this disease (Eguchi et al., 1996; Madrahimov et al., 2006; Detry et al., 2013). Indeed, subtotal hepatectomy (resection of 92% of the liver mass) is a reproducible model that induces death by intracranial hypertension and brain herniation and presents a therapeutic window for assessing new therapy strategies (Eguchi et al., 1996; Detry et al., 2013; Cittolin-Santos et al., 2019). We performed in this study subtotal hepatectomy in rats and evaluated astrocyte morphology, neurochemical parameters, redox homeostasis and brain energy metabolism. The objective of the present study is to elucidate early cerebral metabolic disturbances in acute HE.

## Materials and Methods

### Reagents

All chemicals were purchased from Sigma-Aldrich (St. Louis, MO, USA). Glucose-D, [^14^C(U)] (ARC0122H) and Lactic acid, L-[11-^4^C] sodium salt were purchased from American Radiolabeled Chemicals, Inc., (St. Louis, MO, USA). Glutamic acid, L-[^14^C(U)] (#NEC290E250UC) and Optiphase “Hisafe” 3 (−437) scintillation liquid were purchased from PerkinElmer (Boston, MA, USA). Protein quantification was performed with the BCA Protein Assay kit from Thermo Fisher Scientific (#23227, Rockford, IL, USA), using bovine serum albumin (BSA) as standard.

### Animals

Experiments were performed on 90-day-old male Wistar rats obtained from the Central Animal House of the Department of Biochemistry, ICBS, at the Universidade Federal do Rio Grande do Sul, Porto Alegre, RS, Brazil. The animals were maintained in a 12:12 h light/dark cycle (lights on 07:00–19:00 h) and in an air-conditioned constant temperature (22 ± 1°C) colony room with free access to water and standard commercial chow (SUPRA, Porto Alegre, RS, Brazil). The experimental protocol was approved by the Ethics Committee for Animal Research of the Universidade Federal do Rio Grande do Sul, Porto Alegre, Brazil, under the project number 29468, and followed the NIH Guide for the Care and Use of Laboratory Animals (NIH publication 85-23, revised 1996). All efforts were made to minimize the number of animals used and their suffering.

### Surgical Procedure

Subtotal hepatectomy was performed according to previous descriptions, with minor modifications (Kieling et al., 2012; Detry et al., 2013; Cittolin-Santos et al., 2019). Anesthesia was induced and maintained with 3% isoflurane and an oxygen flow of 0.8 L/min during the whole procedure. The animals were placed on a warmed operating table and a midline laparoscopy was performed to expose the liver. Hepatic ligaments were resected and then pedicles of the anterior lobes were ligated with a 4–0 silk thread to interrupt the blood flow to allow lobe resection. The same procedure was then performed on the right lobes. Only the omental lobes (8% of the liver mass) remained functional. The abdominal wall was sutured with 4–0 nylon thread. Sham group was submitted to the same protocol, except none of the liver lobes pedicles were ligated nor resected.

The animals received intramuscular lidocaine in the abdominal wound to reduce postoperative pain and were kept in a warmed box until full recovery from the anesthesia before being returned to their home cages. Animals had free access to 20% glucose in the drinking water during the whole experiment. Also, three glucose injections of the same glucose solution were administered (2 ml/kg, i.p.) after the surgery to avoid hypoglycemia at the time marks 0, 6 and 12 h.

### Tissue Preparation

Twenty-four hours after the surgery, the rats were euthanized by decapitation, and blood was immediately collected in heparinized tubes. The blood samples were then centrifuged at 2,500× *g* for 10 min at 20°C to yield the plasma fraction for subsequent biochemical analyses. Also, the same animals had samples of the cerebral cortex dissected and separated to evaluate the following parameters: (I) astrocytic reactivity; (II) oxygen consumption; (III) metabolic enzyme activities; (IV) substrates oxidation to CO_2_; and (V) redox homeostasis.

### Immunohistochemistry and Astrocyte Morphological Analysis

Immunohistochemistry for glial fibrillary acidic protein (GFAP) positive astrocyte was performed to evaluate morphological parameters. After decapitation, brains were fixed by immersion for 24 h in 4% PFA diluted in phosphate buffer saline (PBS, pH 7.4), cryoprotected through immersion in sucrose solution (gradually, 15% to 30% until sinking) and frozen at −20°C. Coronal brain slices of 30 μm, approximately +2.20 mm rostrally from bregma, were obtained using a cryostat (MEV, SLEE Medical GMBH, Mainz, Germany). Brain slices were post-fixed with 4% PFA-PBS for 15 min, permeabilized in 0.1% Triton X-100 diluted in PBS (PBS-Tx), and then blocked for 1 h with 5% fetal goat serum diluted in PBS-Tx. The samples were incubated for 24 h at 4°C with polyclonal rabbit anti-GFAP (Z0334, 1:500 in PBS-Tx, Dako, Glostrup, Denmark), followed by 2 h incubation with goat anti-rabbit AlexaFluor^®^ 555 sary antibody (1:1,000 in PBS-Tx, Invitrogen, Carlsbad, CA, USA). Images were obtained in Leica TCS SP5 II laser-scanning confocal microscopy and acquired at 8-bit gray-scale (256 gray levels) using the Leica Application Suite Advanced Fluorescence software (Leica Microsystems, Munich, Germany). The Sholl’s mask creation (virtual concentric circles and orthogonal lines) and all analyses were performed using the ImageJ software, a public domain Java Image processing program^1^.

### Plasma Biochemical Parameters Evaluation

Plasma ammonia, glucose, lactate, alanine aminotransferase (ALT) and aspartate aminotransferase (AST) were measured using commercial kits (Lab test, MG, Brazil) and a SpectraMax M5 microplate reader (Molecular Devices, CA, USA; de Assis et al., 2009).

### Preparation of Mitochondrial Fractions

Twenty-four hours after the surgery, cerebral cortex mitochondria were isolated as previously described (Rosenthal et al., 1987) with slight modifications (Mirandola et al., 2008). Immediately after decapitation, the brain was rapidly removed, the cerebral cortex was dissected and placed into an ice-cold isolation buffer containing 225 mM mannitol, 75 mM sucrose, 1 mM EGTA, 0.1% (BSA; fatty acid-free) and 10 mM HEPES, pH 7.2. The tissue was cut into small pieces using surgical scissors and extensively washed to remove the blood and then homogenized with 1.5 ml of isolation buffer. The homogenate was centrifuged for 3 min at 2,000× *g*. After centrifugation, the supernatant was centrifuged again for 8 min at 12,000× *g*. The pellet was resuspended in 1 ml of isolation buffer containing 4 μl of 10% digitonin and centrifuged for 10 min at 12,000× *g*. The final pellet containing the mitochondria was gently washed and suspended in isolation buffer devoid of EGTA, at an approximate protein concentration of 8 mg/ml.

### Determination of Mitochondrial Respiratory Parameters by Oxygen Consumption

Oxygen consumption rate was measured using an OROBOROS Oxygraph-2k (Innsbruck, Austria) in a thermostatically controlled environment (37°C) and magnetically stirred in an incubation chamber (2 ml of standard reaction medium) in respiring medium containing 0.3 M sucrose, 5 mM KH2PO4, 1 mM EGTA, 1 mg/ml BSA, 5 mM 3-[N-morpholino] propane sulfonic acid (MOPS), pH 7.4, using glutamate plus malate (2.5 mM each) as substrates. State 3 respiration was measured after the addition of 1 mM ADP to the incubation medium. To measure resting (state 4) respiration, 1 μg/ml oligomycin A was added to the incubation medium. The respiratory control ratio (RCR: state 3/state 4) was then calculated. The uncoupled respiration was induced by the addition of carbonyl cyanide m-chlorophenyl hydrazone (CCCP, 2 μM). States 3, 4 and uncoupled respiration were calculated as nmol O_2_ consumed/min/mg protein, and the results were expressed as a percentage of control.

### Determination of ATP Concentration

In order to measure brain ATP levels in frontoparietal cortex, animals were euthanized by decapitation and the whole head was immediately submerged in liquid nitrogen. Once the head was utterly frozen, brain tissue was quickly harvested with a hammer and chisel through a median craniectomy, and the brain tissue (while still frozen) was submerged in 200 μl of 0.7 N perchloric acid at 4°C. The samples were then homogenized and centrifuged (16,000× *g* for 10 min at 4°C). The supernatants were neutralized with 4.0 N KOH and clarified with second centrifugation (16,000× *g* for 30 min at 4°C). After the second centrifugation, the supernatants were collected and centrifuged a third time (16,000× *g* for 30 min at 4°C). ATP analysis was performed by HPLC, as previously described (Voelter et al., 1980). Aliquots of 20 μl were applied to a reversed-phase HPLC (Shimadzu, Japan) using a C18 column (Ultra C18, 25 cm × 4.6 mm × 5 μm, Restek Corporation, Bellefonte, PA, USA). The elution was carried out by applying a linear gradient from 100% solvent A (60 mM KH2PO4 and 5 mM of tetrabutylammonium chloride, pH 6.0) to 100% of solvent B (solvent A plus 30% methanol) over a 30-min period (flow rate at 1.4 ml/min). The amounts of purines were measured by absorption at 254 nm. The retention times of standards were used as parameters for identification and quantification.

### Determination of Glutamate Dehydrogenase (GDH) Activity

Glutamate dehydrogenase (GDH) activity was assayed according to Colon et al. (1986). The reaction mixture contained mitochondrial preparations (60 μg of protein), 50 mM triethanolamine buffer, pH 7.8, 2.6 mM EDTA, 105 mM ammonium acetate, 0.2 mM NADH, 10 mM α-ketoglutarate and 1.0 mM ADP. The reduction of NADH absorbance was monitored spectrophotometrically at 340 nm. GDH activity was calculated as nmol NADH/min/mg protein.

### Determination of Malate Dehydrogenase (MDH) Activity

Malate dehydrogenase (MDH) activity was measured according to Kitto et al. (1970). The incubation medium consisted of mitochondrial preparations (1 μg of protein), 10 μM rotenone, 0.1% Triton X-100, 0.14 mM NADH, 0.3 mM oxaloacetate and 50 mM potassium phosphate, pH 7.4. MDH activity was determined following the reduction of NADH fluorescence at wavelengths of excitation and emission of 366 and 450 nm, respectively. MDH activity was calculated as nmol NADH/min/mg protein.

### Determination of α-Ketoglutarate Dehydrogenase (α-KGDH) Complex Activity

The α-KGDH complex activity was evaluated according to Lai and Cooper (1986) and Tretter and Adam-Vizi (2004) with some modifications. The incubation medium contained mitochondrial preparations (250 μg of protein), 1 mM MgCl_2_, 0.2 mM thiamine pyrophosphate, 0.4 mM ADP, 10 μM rotenone, 0.2 mM EGTA, 0.12 mM coenzyme A-SH, 1 mM α-ketoglutarate, 2 mM NAD^+^, 0.1% Triton X-100 and 50 mM potassium phosphate, pH 7.4. The reduction of NAD^+^ was recorded at wavelengths of excitation and emission of 366 and 450 nm, respectively. The α-KGDH activity was calculated as nmol NADH/min/mg protein.

### Determination of Citrate Synthase (Kaplan et al., 2015) Activity

CS activity was measured according to Shepherd and Garland (1969), by determining 5,5-dithio-bis (2-nitrobenzoic acid; DTNB) reduction at *λ* = 412 nm. The incubation medium contained mitochondrial preparations (2 μg of protein), 5 mM potassium phosphate buffer, pH 7.4, 300 mM sucrose, 1 mM EGTA, 0.1% BSA, 5 mM MOPS, 0.1% Triton X-100, 0.1 mM DTNB, 0.1 mM acetyl-CoA and 0.2 mM oxaloacetate. CS activity was calculated as nmol TNB/min/mg protein.

### Determination of Succinate Dehydrogenase (SDH) Activity

Succinate dehydrogenase (SDH) activity was measured according to Fischer et al. (1985) by determining 2,6-dichloroindophenol (DCIP) reduction at *λ* = 600 nm. The incubation medium contained tissue supernatant (30 μg of protein), 40 mM potassium phosphate buffer pH 7.4, 16 mM sodium succinate, 4 mM sodium azide, 7 μM rotenone, 8 μM DCIP and 1 mM phenazine methosulfate. SDH activity was calculated as nmol reduced DCIP/min/mg protein.

### Redox Assays

#### Reactive Oxygen Species (ROS) Levels

To assess ROS levels, DCFH-DA was used as a probe (LeBel et al., 1992). An aliquot of the parietal cortex homogenate (100 μg–30 μl) was incubated with DCFH-DA (100 μM) at 37°C for 30 min. The formation of fluorescent DCF was monitored at excitation and emission wavelengths of 488 and 525 nm, respectively, using a fluorescence spectrophotometer. ROS contents were quantified using a DCF standard curve. The results are expressed as nmol DCF formed/mg protein.

#### Antioxidant Enzymes Activities

Superoxide dismutase (Ulm et al., 2007; EC 1.15.1.1) activity was assessed on parietal cortex samples by quantifying the inhibition of superoxide-dependent adrenaline auto-oxidation at 480 nm, as previously described, and the results were expressed as units SOD/mg protein (Boveris, 1984). Glutathione peroxidase (GSH-Px; EC 1.11.1.9) activity was measured according to Wendel (1981). One unit of GSH-Px activity was defined as 1 μmol NADPH consumed/min and the specific activity is expressed as units/mg protein.

### Substrates Oxidation to ^14^CO_2_

Cerebral cortex slices (300 μm, 100–120 mg) were obtained as described above, transferred into flasks and pre-incubated in Dulbecco’s buffer for 30 min. Before incubation with substrates, the reaction medium was gassed with a 95% O_2_:5% CO_2_ mixture for 30 s. Slices were incubated in 1 ml of Dulbecco’s buffer containing either: (i) 5 mM D-Glucose + 0.2 μCi D-[^14^C(U)]glucose (American Radiolabeled Chemicals, Inc., St. Louis, MO, USA); (ii) 10 μM L-glutamic Acid + 0.2 μCi L-[^14^C(U)] Glutamate (PerkinElmer Boston, MA, USA); and (iii) 10 μM sodium L-lactate + 0.2 μCi L-[U-^14^C]lactate (American Radiolabeled Chemicals, Inc., St. Louis, MO, USA). Then, flasks containing the slices were sealed with rubber caps and parafilm and incubated at 37°C for 1 h in a Dubnoff metabolic shaker (60 cycles/min) as described previously (Dunlop et al., 1975; Ferreira et al., 2007). The incubation was stopped by adding 0.2 ml of 50% trichloroacetic acid (TCA) through the rubber cap into the flask while 0.1 ml of 2 N NaOH was injected into the central wells. Thereafter, flasks were shaken for an additional 30 min at 37°C to trap CO_2_. Afterward, the content of the central well was transferred to vials and assayed for ^14^CO_2_ radioactivity in a liquid scintillation counter. All the results are expressed as nmol of substrate oxidized per mg of tissue and the initial specific activity of the incubation medium was considered for calculations (Müller et al., 2013).

### Statistical Analysis

The data are expressed as mean ± SEM. All analyses were performed with Prism GraphPad (Version 6.01 for Windows, GraphPad Software, San Diego, CA, USA^2^). Differences among the groups were analyzed by *t*-test with levels of significance below *p* < 0.05 indicated in the following section.

## Results

An initial cohort of 20 animals was operated on and observed to evaluate the efficiency of the surgical procedure and compare it to previous reports (Cittolin-Santos et al., 2019). Our findings were similar to previous studies demonstrating 80% lethality from 30 to 60 h after the surgical procedure (Supplementary Figure S1; Cittolin-Santos et al., 2019). Considering the above stated, a 24-h post-surgery time mark was chosen to collect blood and brain samples, because at this point all animals presented signs of encephalopathy although most were still alive. The second cohort of animals was operated on to obtain samples of blood or cerebral cortex (frontoparietal), and the third cohort of animals were later operated on to perform brain ATP measurement.

### Plasma Biochemical Parameters

In Table 1, we observed that the hepatectomy group presented several plasma alterations that are consistent with ALF, as previously described (Eguchi et al., 1996; Detry et al., 2013; Cittolin-Santos et al., 2019). Hepatectomized animals presented higher levels of ammonia (HEPATEC: 48.4 ± 3.9 vs. SHAM: 22.4 ± 2.1; μmol/L, *p* < 0.001), lactate (HEPATEC: 5.2 ± 1.1 vs. SHAM: 1.5 ± 0.4; mg/dL, *p* < 0.001), ALT (HEPATEC: 61.6 ± 3.1 vs. SHAM: 38.1 ± 1.8 U/L, *p* < 0.001) and AST (HEPATEC: 54.9 ± 4.5 vs. SHAM: 32.6 ± 2.7 U/L, *p* < 0.001) than the controls. The hepatectomy group presented lower levels of glucose (HEPATEC: 71 ± 9 vs. SHAM: 99 ± 8; mg/dL, *p* < 0.05) compared to the sham group.

**Table 1:** Plasma biochemical parameters.

	Sham	Hepatectomy
Ammonia (μmol/L)	22.4 ± 2.1	48.4 ± 3.9***
Glucose (mg/dL)	99 ± 8	71 ± 9*
Lactate (mg/dL)	1.5 ± 0.4	5.2 ± 1.1***
ALT (U/L)	38.1 ± 1.8	61.6 ± 3.1***
AST (U/L)	32.6 ± 2.7	54.9 ± 4.5***

Twenty-four hours after hepatectomy, animals with acute liver failure (ALF) presented higher levels of ammonia (48.4 ± 3.9 vs. 22.4 ± 2.1; μmol/L), lactate (5.2 ± 1.1 vs. 1.5 ± 0.4; mg/dL), ALT (61.6 ± 3.1 vs. 38.1 ± 1.8 U/L) and AST (54.9 ± 4.5 vs. 32.6 ± 2.7 U/L) than the controls. The hepatectomy group presented lower levels of glucose (71 ± 9 vs. 99 ± 8; mg/dL) compared to the sham-operated group. *p < 0.05 and ***p < 0.001 indicate a significant difference from the sham-operated group (*t*-test).

### Immunohistochemistry and Astrocyte Morphological Analysis

**Figure 1:**
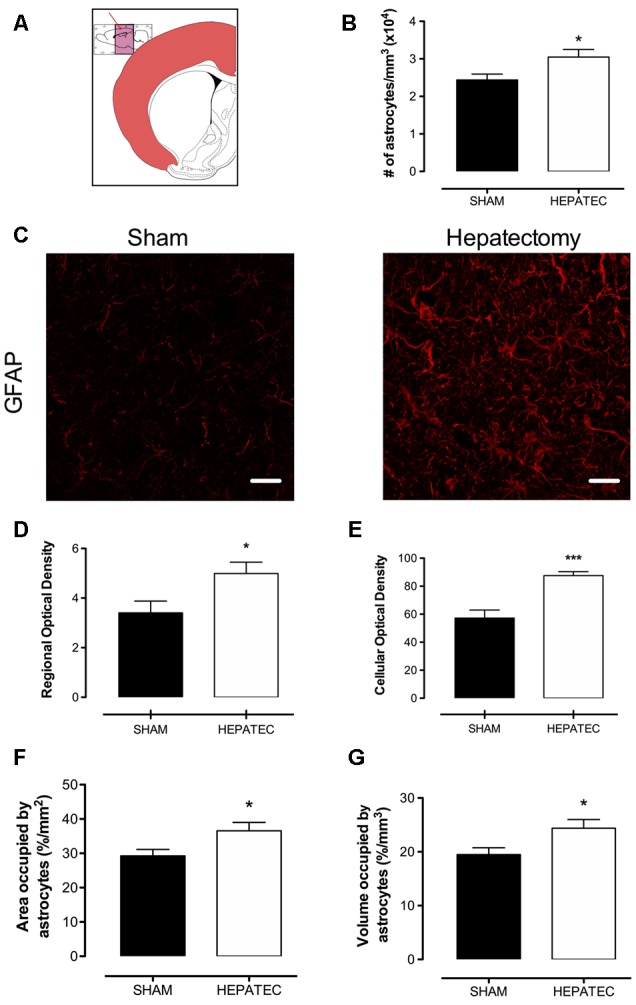
Immunofluorescence for glial fibrillary acidic protein (GFAP). **(A)** represents the region of the brain that was analyzed—parietal cortex; **(B)** represents the number of astrocytes/mm^3^; **(C)** represents immunofluorescence for the GFAP in the parietal cortex. The hepatectomy group presented an increase in regional immunofluorescence for GFAP **(D)** and cellular optical density (87.60 ± 7.39 vs. 57.22 ± 15.21) **(E)**. The area occupied by astrocytes (36.57 ± 2.42 vs. 29.24 ± 1.90) **(F)** and the volume occupied by astrocytes (24.38 ± 1.61 vs. 19.49 ± 1.27) **(G)** were also augmented. Differences between groups were analyzed by *t*-test and are indicated as *p < 0.05 and ***p < 0.001.

**Figure 2:**
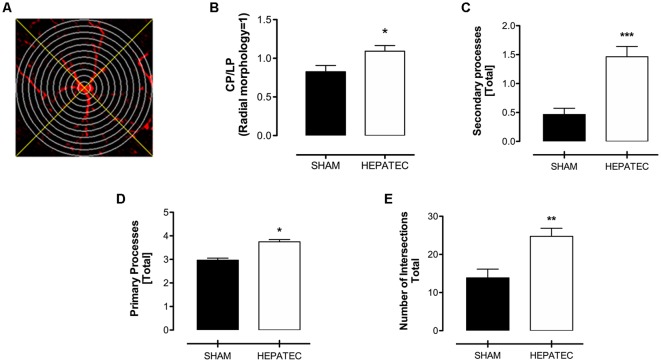
Sholl’s mask creation—virtual concentric circles and orthogonal lines **(A)** and astrocytic processes and intersections. Panel **(B)** expresses the ratio between central processes (McPhail et al., 2010) and lateral processes (LP; 1.09 ± 0.07 vs. 0.83 ± 0.08). Hepatectomy group presented an increase in the size of both cellular primary processes **(C)** (19.68 ± 1.41 vs. 12.90 ± 1.31) and secondary processes **(D)** (1.46 ± 0.43 vs. 0.46 ± 0.26). The number of intersections **(E)** was also increased in animals operated on (24.74 ± 2.13 vs. 13.85 ± 5.62). Differences between groups were analyzed by *t*-test and are indicated as *p < 0.05; **p < 0.01 and ***p < 0.001.

Confocal images of parietal cortex stained with GFAP showed that animals with ALF (Figure 1A) presented an increase in the number of astrocytes/mm^3^ (HEPATEC: 3.05 ± 0.20 vs. SHAM: 2.44 ± 0.16, Figure 1B, *p* < 0.05) as well as in regional optical density (HEPATEC: 4.99 ± 1.12 vs. SHAM: 3.41 ± 1.16, Figures 1C,D, *p* < 0.05) and in cellular optical density (HEPATEC: 87.60 ± 7.39 vs. SHAM: 57.22 ± 15.21, 1E, *p* < 0.001) when compared to the control animals. The area and the volume occupied by astrocytes was also increased when compared to the sham group (HEPATEC: 36.57 ± 2.42 vs. SHAM: 29.24 ± 1.90 and HEPATEC: 24.38 ± 1.61 vs. SHAM: 19.49 ± 1.27, respectively, Figures 1F,G, *p* < 0.05). Nonetheless, the number of brain cells was equal in both groups (data not shown). Regarding cellular morphology (Figure 2A), the astrocytes of hepatectomized animals presented a general increase in the ratio of Central processes/lateral processes (LP; HEPATEC: 0.82 ± 0.03 vs. SHAM: 1.09 ± 0.04, Figure 2B, *p* < 0.05), number of primary processes (HEPATEC: 3.74 ± 0.23 vs. SHAM: 2.97 ± 0.21, Figure 2C, *p* < 0.05) and secondary processes (HEPATEC: 1.46 ± 0.43 vs. SHAM: 0.46 ± 0.26, Figure 2D, *p* < 0.001). Consequentially, the number of intersections between these cellular processes was also significantly increased in animals with ALF when compared to the sham group (HEPATEC: 24.74 ± 2.13 vs. SHAM: 13.85 ± 5.62, Figure 2E, *p* < 0.01). Additional analysis of astrocytic morphology is also expressed in Supplementary Figure S2.

### Oxygen Consumption

**Figure 3:**
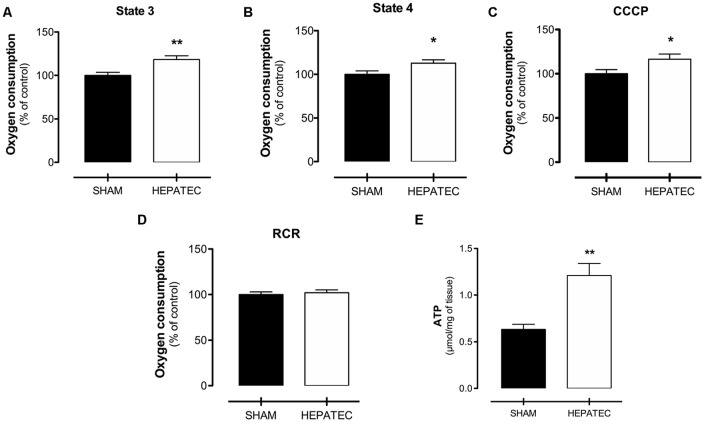
Mitochondrial oxygen consumption. Animals with acute liver failure (ALF) presented higher oxygen consumption in mitochondrial state 3 (118.20 ± 9.58 vs. 100.00 ± 7.90 as % of control and 833.91 ± 93.51 vs. 753.91 ± 79.42 as pmol/s/mg) **(A)**; mitochondrial state 4 (112.80 ± 8.63 vs. 100.00 ± 9.73 as % of control and 121.26 ± 14.68 vs. 111.83 ± 10.88 as pmol/s/mg) **(B)** and CCCP (116.40 ± 13.09 vs. 100.00 ± 10.31 as % of control and 858.81 ± 115.82 vs. 792.22 ± 99.09 as pmol/s/mg) **(C)**. No differences were found in respiratory control ratio (RCR) oxygen consumption (102.10 ± 7.47 vs. 100.00 ± 7.15 as % of control and 6.90 ± 0.50 vs. 6.76 ± 0.48 as pmol/s/mg) **(D)**. Brain ATP was also augmented in animals with ALF (1.20 ± 0.13 vs. 0.63 ± 0.05, **(E)** *p* < 0.05, μmol/mg). **(E)** The results in the figure are expressed as (% of control). Differences between groups were analyzed by *t*-test and are indicated as *p < 0.05 and **p < 0.01.

Mitochondrial oxygen consumption was mostly increased in animals with HE compared to the control group. Indeed, an elevated oxygen consumption level was found in state 3 (HEPATEC: 118.20 ± 9.58 vs. SHAM: 100.00 ± 7.90, Figure 3A, *p* < 0.01), in-state 4 (HEPATEC: 112.80 ± 8.63 vs. SHAM: 100.00 ± 9.73 m, Figure 3B, *p* < 0.05) and in uncoupled respiration (CCCP; HEPATEC: 116.40 ± 13.09 vs. SHAM: 100.00 ± 10.31, Figure 3C, *p* < 0.05). No alterations were found in the RCR (HEPATEC: 102.10 ± 7.47 vs. SHAM: 100.00 ± 7.15, Figure 3D). Results are expressed as a percentage of control.

### ATP Levels

Parietal cortex ATP levels were significantly elevated in animals with ALF (HEPATEC: 1.20 ± 0.13 vs. SHAM: 0.63 ± 0.05, Figure 3E, *p* < 0.05), as expressed in Figure 3E. Results are expressed as μmol/mg of tissue.

### Enzyme Activities of Brain Energy Metabolism

**Figure 4:**
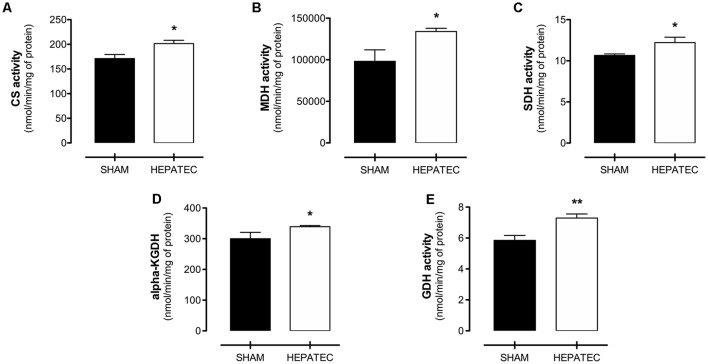
Enzyme activities of brain energy metabolism. Hepatectomy group was found to have an increase in the activity of all analyzed enzymes: **(A)** Citrate synthase (201.05 ± 16.11 vs. 171.10 ± 20.03); **(B)** Malate dehydrogenase (MDH; 133,954 ± 8,690 vs. 98,149 ± 30,939); **(C)** Succinate dehydrogenase (SDH; 12.22 ± 1.46 vs. 10.65 ± 0.39); **(D)** Alpha-ketoglutarate dehydrogenase (α-KGDH; 7.29 ± 0.59 vs. 5.85 ± 0.71); and **(E)** Glutamate dehydrogenase (GDH; 339.1 ± 8.24 vs. 299.90 ± 46.46). The results are expressed as nmol/min/mg of protein. Differences between groups were analyzed by *t*-test and are indicated as *p < 0.05 and **p < 0.01.

Hepatectomized animals presented increased activity in all evaluated metabolic enzymes when compared to the sham group (Figure 4). Enzyme activity in hepatectomized and control group was, respectively: citrate synthase (HEPATEC: 201.05 ± 16.11 vs. SHAM: 171.10 ± 20.03, Figure 4A, *p* < 0.05); MDH (HEPATEC: 133,954 ± 8,690 vs. SHAM: 98,149 ± 30,939, Figure 4B, *p* < 0.05); SDH (HEPATEC: 12.22 ± 1.46 vs. SHAM: 10.65 ± 0.39, Figure 4C, *p* < 0.05); α-KGDH (HEPATEC: 7.29 ± 0.59 vs. SHAM: 5.85 ± 0.71, Figure 4D, *p* < 0.05); and GDH (HEPATEC: 339.1 ± 8.24 vs. SHAM: 299.90 ± 46.46, Figure 4E, *p* < 0.01). The results are expressed as nmol of substrate/min/mg of protein.

### Substrates Oxidation to ^14^CO_2_

**Figure 5:**
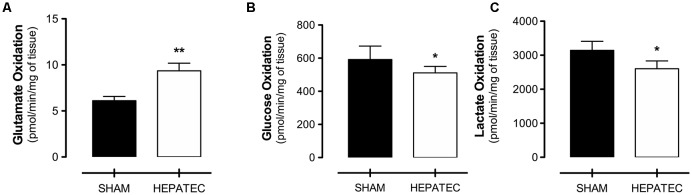
Substrate oxidation to CO_2._ Animals submitted to hepatectomy presented elevated glutamate oxidation (9.36 ± 0.82 vs. 6.11 ± 0.45) **(A)**. However, the hepatectomy group had lower oxidation of glucose (511.30 ± 38.86 vs. 591.80 ± 80.97) **(B)** and lactate (2,602.0 ± 228.90 vs. 3,142.0 ± 266.50) **(C)** when compared to control animals. Results are expressed in pmol/min/mg of tissue. Differences between groups were analyzed by *t*-test and are indicated as *p < 0.05 and **p < 0.01.

Animals submitted to hepatectomy presented an increase in glutamate oxidation to ^14^CO_2_ (HEPATEC: 9.36 ± 0.82 vs. SHAM: 6.11 ± 0.45, Figure 5A, *p* < 0.01) while presenting lower oxidation to ^14^CO_2_ of glucose (HEPATEC: 511.30 ± 38.86 vs. SHAM: 591.80 ± 80.97, Figure 5B, *p* < 0.05) and lactate (HEPATEC: 2,602.0 ± 228.90 vs. SHAM: 3,142.0 ± 266.50, Figure 5C, *p* < 0.05). The results are expressed as pmol of substrate/min/mg of tissue.

### Redox Assays

**Figure 6:**
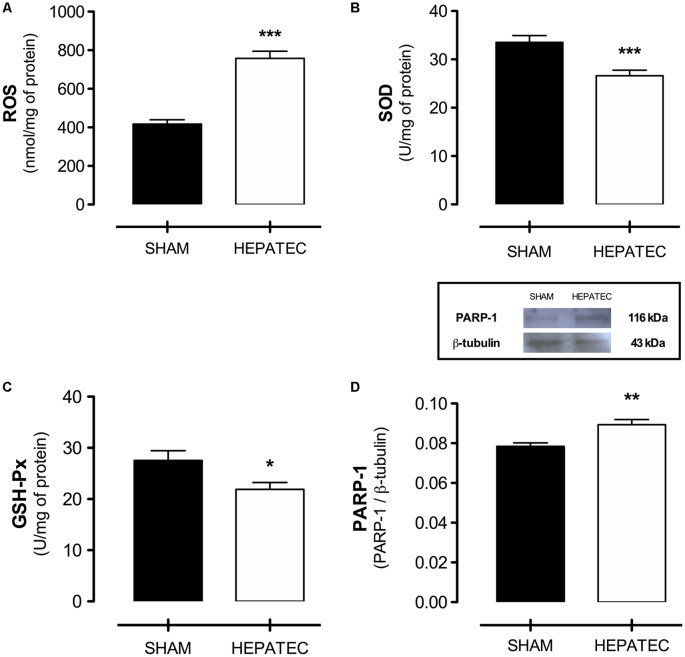
Redox homeostasis and PARP-1 expression. The hepatectomy group presented elevated levels of reactive oxygen species (ROS) **(A)** (757.80 ± 36.82 vs. 417.00 ± 22.54) as well as decreased SOD **(B)** (26.62 ± 1.19 vs. 33.54 ± 1.38) and GSH-Px **(C)** (21.90 ± 1.32 vs. 27.55 ± 1.89) activity. An increased expression in PARP-1 content was also encountered in this group of animals **(D)** (0.089 ± 0.002 vs. 0.078 ± 0.001). ROS are expressed as nmol/mg of protein; the enzyme activity is expressed as U/mg of protein and PARP-1 expression as PARP-1/b-tubulin ratio. Differences between groups were analyzed by *t*-test and are indicated as *p < 0.05; **p < 0.01 and ***p < 0.001.

Several alterations in the redox homeostasis were induced by hepatectomy. Acute HE presented elevated levels of ROS (HEPATEC: 757.80 ± 36.82 vs. SHAM: 417.00 ± 22.54, Figure 6A, *p* < 0.001). Accordingly, it caused a decrease in the activity of two essential antioxidant enzymes: SOD (HEPATEC: 26.62 ± 1.19 vs. SHAM: 33.54 ± 1.38, Figure 6B, *p* < 0.001) and GSH-Px (HEPATEC: 21.90 ± 1.32 vs. SHAM: 27.55 ± 1.89, Figure 6C, *p* < 0.05). PARP-1 immunocontent in the cerebral cortex of hepatectomized animals was elevated when compared to the sham group (HEPATEC: 0.089 ± 0.002 vs. SHAM: 0.078 ± 0.001, respectively, Figure 6D, *p* < 0.01).

## Discussion

ALF is a meaningful and potentially life-threatening syndrome that is caused by liver damage and substantial neurotoxin accumulation in the brain, such as ammonia (Bernal, 2017). Indeed, blood ammonia elevation plays an essential role in the development of encephalopathy and leads to glutamatergic excitotoxicity, oxidative stress and astrocytic dysfunction (Ciećko-Michalska et al., 2012; Butterworth, 2015). In the present study, we used an experimental model of ALF induced by subtotal hepatectomy to investigate astrocyte reactivity, brain redox status, energy metabolism and mitochondrial function in rodents. To our knowledge, this is the first study describing evidence of a brain hypermetabolic state induced by ALF, as previous similar results had been found only using cell cultures or acute ammonium intoxication models (Kosenko et al., 1994; Xue et al., 2010). The hypermetabolic state involved the increase in brain oxygen consumption and activities of mitochondrial enzymes, elevated ATP levels, and increased glutamate oxidation. We also postulate a new link between the hypermetabolic state of HE and the increase of ROS with astrocytic reactivity, suggesting a new understanding of the early (24 h) brain metabolic profile in acute HE.

For this discussion, it is crucial to emphasize that hepatectomy causes up to 80% lethality in the interval from 30 to 60 h after surgery (Supplementary Figure S1), as demonstrated in the previous work of our group (Cittolin-Santos et al., 2019). Therefore, the 24-h time mark after surgery was chosen to harvest blood and brain samples as most animals were still alive and already showing marked signs of HE such as ataxia, lethargy and diminished response to pain (Cauli et al., 2008). At this point, animals also presented elevated plasma liver enzymes and alterations in blood glucose, lactate and ammonia levels (Table 1) that are well described in this animal model of ALF (Eguchi et al., 1996; Detry et al., 2013; Fusco et al., 2013; Cittolin-Santos et al., 2019).

As mentioned before, elevated intracranial pressure is an essential contributor to HE’s high lethality rates (Larsen and Wendon, 2008) and understanding the mechanisms that contribute to this process may be vital in establishing better patient care. Previous studies with magnetic resonance imaging performed in humans with ALF have shown a reduction in the brain’s apparent diffusion coefficient, indicating an increase in cellular volume and brain edema (Keiding and Pavese, 2013). Indeed, ALF induces an accumulation of glutamine in the astrocyte in an attempt to detoxify ammonia which has been linked to astrocyte swelling and dysfunction due to glutamine’s osmolyte effect (Scott et al., 2013; Rama Rao et al., 2014). In parallel, astrocytic reactivity may be a defense mechanism to modulate brain homeostasis by increasing astrocytic workload, and it may contribute to the elevation of brain pressure due to increased cellular volume. This process has been described in several cerebral diseases, including experimental and human HE (Pilbeam et al., 1983; Kimura et al., 2008). In this manuscript, we describe severe astrocytic morphological changes that characterize a state of diffuse astrocytic reactivity. Indeed, we encountered a significant increase in cellular optical density and astrocytic volume consequential to the increase of astrocytic processes. No alterations were found in the number of astrocytes, indicating that the increase in GFAP density was due to the proliferation in size and number of astrocytic processes. Previous work using the same ALF experimental model that our study used demonstrated an increase in the intracranial pressure following hepatectomy in rats (Detry et al., 2013). Although we did not directly measure intracranial pressure, we demonstrate a state of diffuse astrocytic growth early in the development of HE which could indicate that cytotoxic edema may not be the only mechanism involved in the expansion of the total astrocytic volume and resulting increased intracranial pressure.

Regarding mitochondrial function and brain bioenergetics, there are controversial data about the influence of hyperammonemia on TCA enzymatic activity and energy production (Schousboe et al., 2014; Heidari, 2019). Classic *in vitro* studies has shown that ammonium intoxication inhibits critical enzymes in brain energy metabolism (Bessman and Bessman, 1955). On the other hand, excess ammonium leads to a disturbance in glutamatergic homeostasis which has been linked to increased glycolysis (increased activity of phosphofructokinase and aldolase) and increased activity of TCA enzymes (Zwingmann et al., 2003). Normal brain ATP levels and TCA activity have been described in hyperammonemia and soon after experimental liver devascularization (Holmin et al., 1983; Fitzpatrick et al., 1989; Mans et al., 1994). In our current study, we found that the enzymatic activity of pathways involved in bioenergetics metabolism and oxygen consumption were elevated 24 h after hepatectomy, which was accompanied by elevated brain cortical ATP levels and thus indicating a significant alteration in the energy metabolism homeostasis. Since the alterations in brain bioenergetics follow the rapid rise in ammonium levels due to liver insufficiency, we hypothesize that the astrocytes require high levels of energy consumption to remove glutamate from the synaptic cleft and metabolize it in the TCA cycle. Thus, the increase in oxygen consumption and enzymatic activity could be a reactive mechanism of the neural cells trying to provide enough energy to enable the neural tissue to respond to brain injury, which characterizes a hypermetabolic state. This effect, however, is probably time-dependent, occurring only in the early stages (acute phase) of HE. Indeed, previous evaluations of brain ATP levels found that early HE presented mild elevations of brain ATP 4 h after total hepatectomy (Holmin et al., 1983) while ATP measurement of animals with chronic exposure to high ammonia levels (4 weeks after bile duct ligation) have demonstrated decreased ATP levels (Dhanda et al., 2018).

As stated above, astrocytes are involved in the progression of HE and some authors even propose that HE is primarily a glyopathy (Norenberg, 1996; El Khiat et al., 2019) as these cells are responsible for most of the ammonium and glutamate detoxification. This process, however, consumes a significant amount of glucose to produce glutamine and absorb two ammonia molecules which can lead to a decrease in glucose oxidation to CO_2_. Indeed, Sibson et al. (2001) demonstrated that up to 80% of brain glucose is utilized for ammonia detoxification by glutamine formation in rats with hyperammonemia, while healthy animals utilize around 30% of glucose in this process (Sibson et al., 2001). In our study, both glucose and lactate oxidation to CO_2_ decreased in animals with HE (Figure 5). Nonetheless, several reports of experimental models with acute rises in ammonia levels failed to demonstrate a brain energy deficit (Lin and Raabe, 1985; Fitzpatrick et al., 1989; Mans et al., 1994), and our data indicate elevated mitochondrial oxygen consumption as well as no ATP deficit. This could mean that some compensatory mechanisms are activated during hyperammonemia to sustain energy balance. Johansen et al. (2007) discuss the role of branched-chain amino acids in the reposition of carbon skeletons and glutamate production under HE. In this study, we found that the oxidation of glutamate to CO_2_ was significantly increased in animals 24 h after hepatectomy (Figure 5). We propose that the increase in ammonia levels due to ALF augments the utilization of glucose for glutamine production contributing to the decrease of glucose oxidation to CO_2_. Moreover, the increased glutamate oxidation could, in part, contribute to sustaining brain energy homeostasis as well as removing excess glutamate from the synaptic cleft and potentially attenuating the glutamatergic excitotoxicity and NMDA overstimulation.

Ammonium accumulation in the central nervous system, as described in experimental models of ammonium intoxication and ALF, causes overstimulation of NMDA receptors (Montana et al., 2014; Oja et al., 2017; Dabrowska et al., 2018). This happens both by an increase in extracellular glutamate levels and by direct ammonium activation of NMDA receptors. The NMDA receptor overstimulation is a crucial factor in the development of oxidative stress (Sathyasaikumar et al., 2007; Cittolin-Santos et al., 2017) due to the increase of calcium influx into the cell, which in turn increases ROS production (Hermenegildo et al., 2000; Montes-Cortes et al., 2019). The hepatectomy group presented elevated levels of ROS and decreased activity of SOD and GSH-Px (Figures 6B,C). Similar results regarding SOD activity were previously described in acute ammonia intoxication in rats by our group and others (Kosenko et al., 1998; Görg et al., 2013; Cittolin-Santos et al., 2017). This means that ammonia may cause an imbalance in brain redox status through antioxidant enzymes inhibition as well as glutamatergic overstimulation. Indeed, our group and others have shown that by modulating glutamatergic excitotoxicity there is a normalization of brain redox status and a decrease in lethality under acute ammonia intoxication (Cauli et al., 2014; Paniz et al., 2014; Cittolin-Santos et al., 2017).

Poly (ADP-ribose) polymerase 1 (PARP-1) is a nuclear enzyme involved in critical cellular processes such as DNA repair and cell death (Jubin et al., 2016). The enzymatic activity of PARP-1 is stimulated significantly in the presence of a wide range of activators like damaged DNA, nucleosomes and various protein-binding partners (Eustermann et al., 2011; Langelier et al., 2011). It is noteworthy that oxidative stress induces DNA damage in neural cells (Guo et al., 2013; Narciso et al., 2016) which may act as the signal to activate PARP-1. PARP-1 upregulation has already been linked to increased lethality in experimental models of ALF induced by acetaminophen intoxication, just as PARP-1 inhibition was related to a diminished lethality rate (Dönmez et al., 2015). Considering the above stated and the elevated levels of ROS presented in this manuscript, we hypothesize that oxidative stress is an early event in the development of acute HE that may induce DNA genic activation and PARP-1 upregulation in ALF.

Understanding the complexity of brain metabolic alterations, glial reactivity and cellular dysfunction are the first steps for the development of new treatment strategies for HE due to ALF. In this work, we described several astrocytic alterations that characterize a state of astrocytic reactivity, here observed as an increase in the astrocytic volume due to the proliferation of cellular processes that are known to take part in the pathophysiology of acute HE. We also associated these glial morphological alterations with significant brain metabolic abnormalities such as redox imbalance, increased brain energy metabolism (increased oxygen consumption and enzymatic activities), increased brain ATP levels as well as alterations in substrate oxidation.

Furthermore, as discussed above, glutamine production for ammonia detoxification utilizes an increased amount of glucose in animals with HE. Thus, we propose that the increase in glutamate oxidation may contribute to sustaining brain ATP levels in the early stages of HE. We also found that the brain hypermetabolic state is associated with imbalances in redox homeostasis and upregulation of PARP-1. Finally, we bring new evidence to the literature regarding the association between astrocytic reactivity, oxidative stress and alterations in brain mitochondrial metabolic and in substrate oxidation under experimental ALF.

## Data Availability Statement

All datasets generated for this study are included in the article/Supplementary Material.

## Ethics Statement

The animal study was reviewed and approved by Ethics Committee for Animal Research of the Universidade Federal do Rio Grande do Sul (29468), Porto Alegre, Brazil.

## Author Contributions

PG and GC-S were responsible for the design, acquisition, analysis, interpretation, drafting, and approval of the final version of the manuscript. LM-M, MG, YN, GSL, DM, JS and FF were responsible for acquisition, analysis, interpretation, and approval of the final version of the manuscript. MW and GL were responsible for interpretation, drafting, critical revision, and approval of the final version of the manuscript. DS and AA were responsible for the design, interpretation, drafting, critical revision, and approval of the final version of the manuscript.

## Conflict of Interest

The authors declare that the research was conducted in the absence of any commercial or financial relationships that could be construed as a potential conflict of interest.

## Footnotes

^1^http://rsb.info.nih.gov/ij/

^2^www.graphpad.com

## Supplementary Material

FIGURE S1 Experimental protocol and subtotal hepatectomy lethality. Panel **(A)** represents the surgical protocol and time mark of 24 h post-surgery for sample harvesting. Panel **(B)** represents the overall mortality and of animals after the hepatectomy. The animals operated on presented 80% lethality within the first 30–60 h after the procedure.

FIGURE S2 Astrocytic processes and intersections. The hepatectomy group presented an increase in the number of **(A)** central primary processes (1.75 ± 0.20 vs. 1.23 ± 0.08); **(C)** central secondary processes (0.76 ± 0.08 vs. 0.19 ± 0.08); and **(D)** lateral secondary processes (0.70 ± 0.14 vs. 0.27 ± 0.06). The number of **(B)** lateral primary processes was equal in both groups (1.99 ± 0.17 vs. 1.74 ± 0.10). The number of central **(E)** and lateral **(F)** intersections was also increased in animals with acute liver failure (11.79 ± 2.19 vs. 5.90 ± 0.98 and 13.42 ± 1.70 vs. 7.56 ± 1.44, respectively). Differences between groups were analyzed by *t*-test and are indicated as **p* < 0.05; ***p* < 0.01 and ****p* < 0.001.
