# Supplementary figures and images for: Acute Liver Failure Induces Glial Reactivity, Oxidative Stress and Impairs Brain Energy Metabolism in Rats
Source: Front Mol Neurosci. 2020 Jan 10;12:327. doi: 10.3389/fnmol.2019.00327 (PMC6968792; doi:10.3389/fnmol.2019.00327)

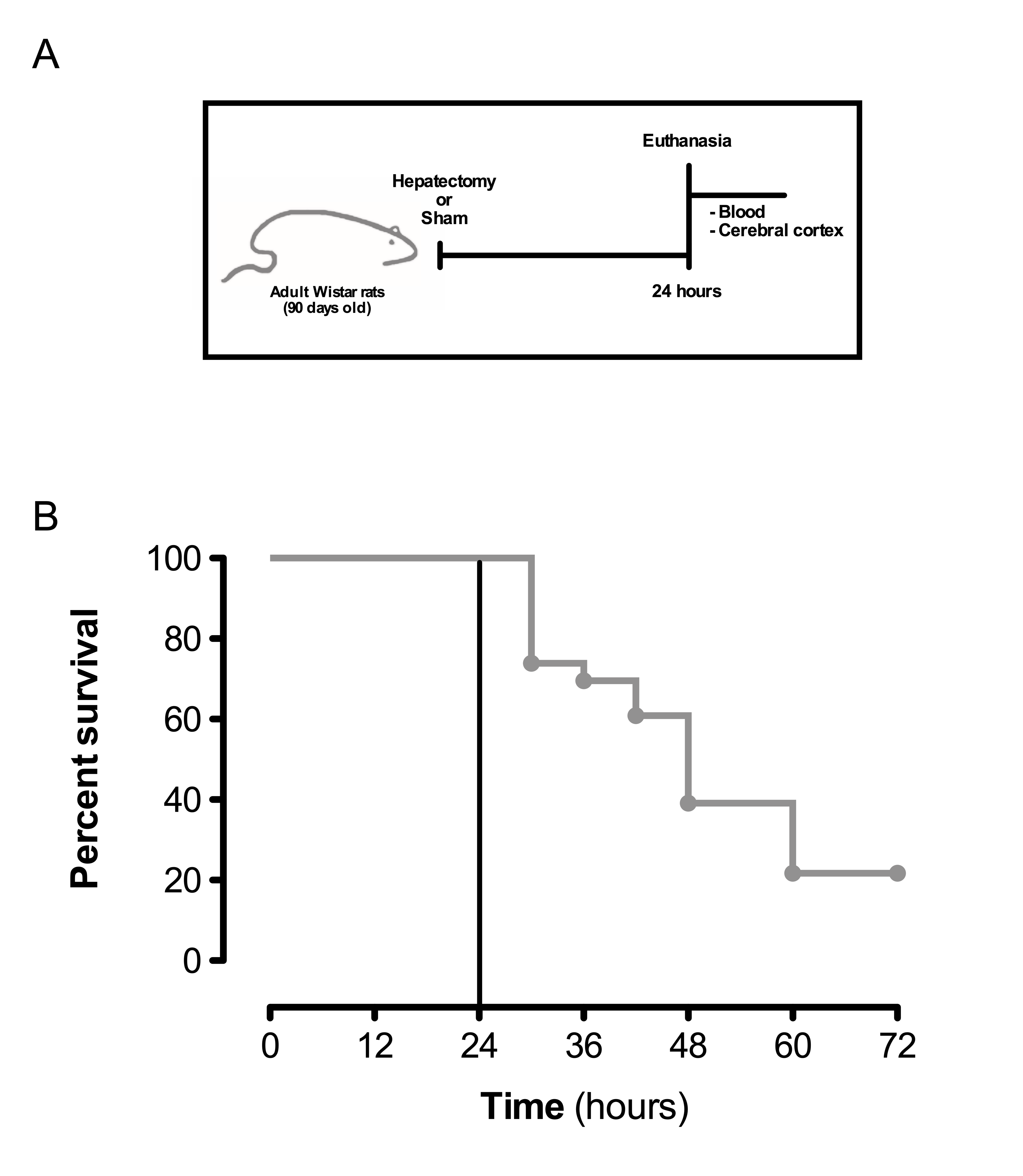

Supplement: FIGURE S1 — Experimental protocol and subtotal hepatectomy lethality. Panel (A) represents the surgical protocol and time mark of 24 h post-surgery for sample harvesting. Panel (B) represents the overall mortality and of animals after the hepatectomy. The animals operated on presented 80% lethality within the first 30–60 h after the procedure. [file Image_1.TIFF]

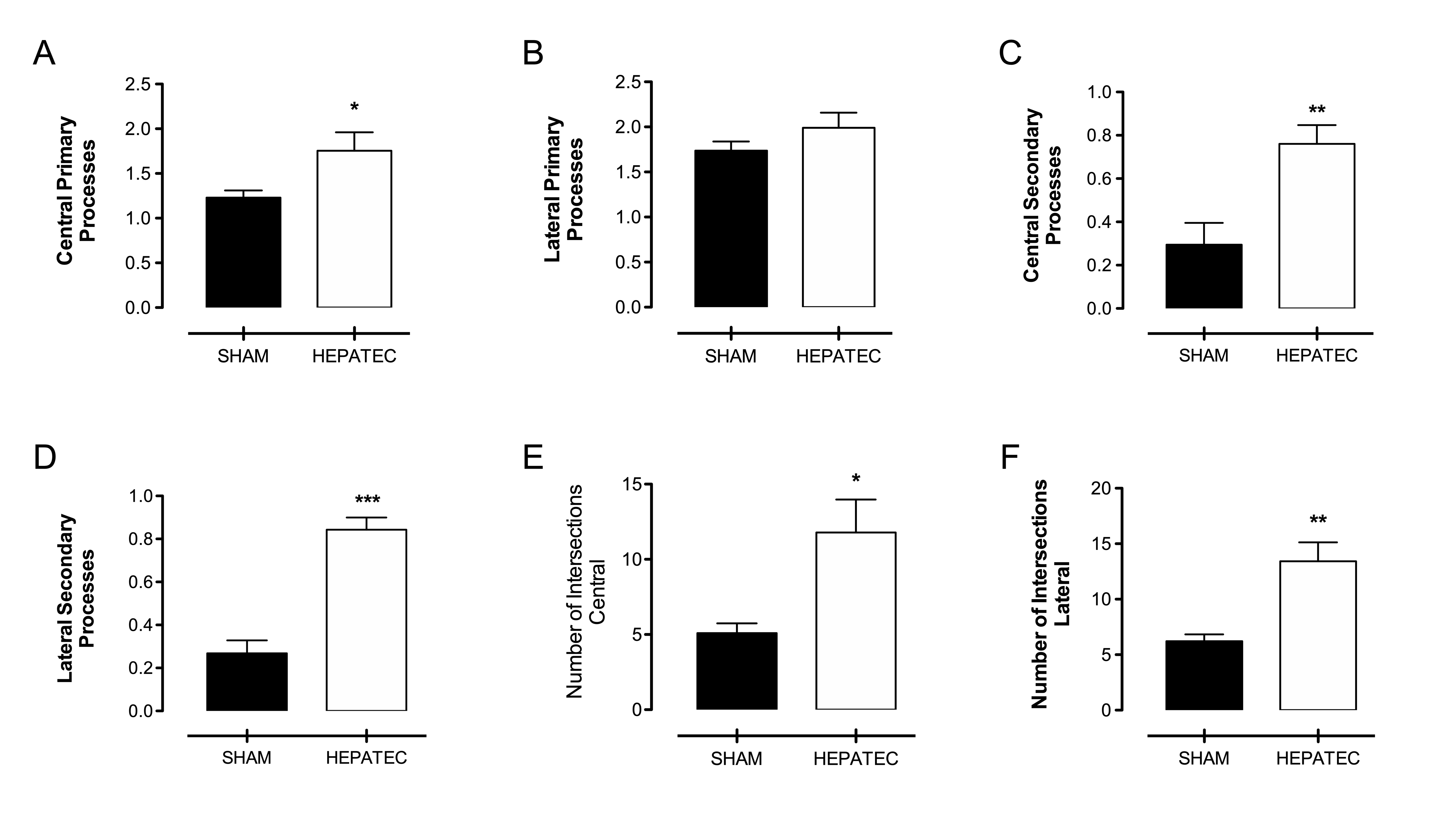

Supplement: FIGURE S2 — Astrocytic processes and intersections. The hepatectomy group presented an increase in the number of (A) central primary processes (1.75 ± 0.20 vs. 1.23 ± 0.08); (C) central secondary processes (0.76 ± 0.08 vs. 0.19 ± 0.08); and (D) lateral secondary processes (0.70 ± 0.14 vs. 0.27 ± 0.06). The number of (B) lateral primary processes was equal in both groups (1.99 ± 0.17 vs. 1.74 ± 0.10). The number of central (E) and lateral (F) intersections was also increased in animals with acute liver failure (11.79 ± 2.19 vs. 5.90 ± 0.98 and 13.42 ± 1.70 vs. 7.56 ± 1.44, respectively). Differences between groups were analyzed by t-test and are indicated as *p < 0.05; **p < 0.01 and ***p < 0.001. [file Image_2.tiff]
